# Transformation of non-water sorbing fly ash to a water sorbing material for drought management

**DOI:** 10.1038/s41598-020-75674-6

**Published:** 2020-10-29

**Authors:** Abhisekh Saha, Sreedeep Sekharan, Uttam Manna, Lingaraj Sahoo

**Affiliations:** 1 Department of Civil Engineering, Indian Institute of Technology, Guwahati, Assam India; 2 Department of Chemistry and Center for Nanotechnology, Indian Institute of Technology, Guwahati, Assam India; 3 Department of Biosciences and Bioengineering, Indian Institute of Technology, Guwahati, Assam India

**Keywords:** Engineering, Climate-change impacts, Environmental chemistry

## Abstract

Securing water in the soil through suitable amendments is one of the methods for drought management in arid regions. In this study, a poor water sorbing fly ash was transformed into a high water-absorbing material for improving soil water retention during the drought period. The fly ash water absorbent (FAWA) exhibited high water-absorbing capacity (WAC) of 310 g/g at par with commercially available superabsorbent hydrogel (SAH). The FAWA showed excellent re-swelling behavior for more than eight alternate wetting–drying cycles. The WAC of FAWA was sensitive to salt type, pH, and ionic strength of the solution. At maximum salinity level permitted for plant growth, the WAC of FAWA was 80 g/g indicating its suitability for drought management. There was only a marginal WAC variation in the range of pH (5.5–7.5) considered most suitable for plant growth. The drying characteristics of FAWA amended soil exhibited an increase in desaturation time by 3.3, 2.2, and 1.5 times for fine sand, silt loam, and clay loam, respectively. The study demonstrates the success of using a low rate of FAWA for drought management with the advantage of offering a non-toxic and eco-friendly solution to mass utilization of industrial solid waste for agricultural applications.

## Introduction

Drought is a major challenge to agriculture by imposing a drastic reduction in crop yield worldwide. Approximately 70% of globally available water is utilized in agriculture, and 60% of world food is produced in rainfed soils^1^. In this context, plant water deficit would seriously risk agriculture in arid and semi-arid regions^2,3^. A decline in food production may result as a consequence of water deficit stress resulting from frequent drought condition^4,5^. This indicates a need to find solutions to mitigate the negative impact of drought on crop production. There exist different drought management strategies, such as early warning systems through monitoring climate data, vulnerable zone identification, and water management policies (conservation of rainwater and streamflow diversion)^6^. Along with these conventional methods, various unconventional measures are also required to reduce the negative influence of drought and increase crop productivity. Soil amendment using high water-absorbing material offers smart solutions to retain soil water during irrigation, for subsequent supply to plant root zone, which can be fine-tuned depending on a specific plant’s needs and water retention capacity of the soil.

Crosslinked superabsorbent hydrogel (SAH) can absorb and retain a large quantity of water and solute molecules in a swollen state, where various hydrophilic groups (e.g., carboxyl groups, amino groups, hydroxyl groups, etc.) attached to the polymeric backbone can readily interact with water molecules^7–11^. The crosslinking makes the polymer insoluble in water and form a gel type of material, which can store water within them^12,13^. The commercially available SAH for horticulture has a water-absorbing capacity (WAC) of 100–300 g/g^14–16^. Due to high WAC, these polymers are receiving much attention in the hygiene industry, drug delivery, food storage, wastewater treatment, biosensors, and tissue engineering^17,18^. The use of SAH as agricultural soil amendment was recommended by the United States Department of Agriculture (USDA) in the 1960s. However, most of the commercial SAHs are “fully synthetic copolymer”, which are synthesized by copolymerizing acrylic acid and acrylamide in the presence of a cross-linker^19^. One inherent limitation of the fully synthetic copolymer is the high production cost. The other type of SAH is “graft copolymer,” where natural materials such as starch, cellulose, clay, chitosan are linked with a hydrophilic polymeric chain^7,20–23^. These grafted copolymers possess high WAC, salt-resistivity, and higher mechanical stability as compared to the fully synthetic polymer (Table 1). For drought management applications, there is a need to synthesize eco-friendly, hybrid copolymer (synthetic + natural polymers), utilizing waste material to minimize the overall cost of production.

**Table 1:** Comparison of various properties of synthetic and hybrid SAH.

Properties	Synthetic SAH	Hybrid SAH
Composition	Comprise of purely synthetic monomer unit (such as acrylic acid, acrylamide)	Natural materials (such as starch, cellulose, clay, chitosan) linked with synthetic monomer unit
Water absorbing capacity (WAC)	WAC ranges between 100 and 300 g/g of SAH	WAC can be as high as 1000 g/g of SAH
Salt sensitivity	Synthetic SAH highly sensitive to the salt solutions	Addition of natural material in polymer network improves the salt sensitivity of SAH
Biodegradability	Lower rate of degradation	Degradation rate is higher due to presence of natural materials
Eco-compatibility	Not very eco-compatible due to the synthetic material	Excellent eco-compatibility
Cost	Higher production cost due to use of pure chemicals	Production cost is lower due to incorporation of low-cost natural materials/waste material
Field of application	Wound dressing, Hygienic applications	Agriculture, horticulture, dryland farming

Fly ash (FA), a waste residue from the thermal power plant, is an amorphous ferro-aluminosilicate very similar to soil^24,25^. In India, around 40% of FA finds its application in the cement industry and various other infrastructural projects, and the remaining percentage remains unutilized^26^. The FA is transported through a pipe in slurry form and disposed of in the ash ponds. These ash ponds not only utilize a large area of usable land but also have adverse effects on the environment. Several past studies have shown that FA can be helpful for plant growth when mixed with soil in optimum quantity^27,28^. It contains various essential plant nutrients, i.e., macronutrients including P, K, Ca, Mg and S, and micronutrients like Fe, Mn, Cu, B, and Mo^26^. It can be noted that FA may contain heavy metals such as Mn, Zn, Cu, Pb, Cr, and Cd, depending on the source of the parent coal^29^. However, several past studies have highlighted that the leaching of heavy metals from FA remains well below the recommended value up to an amendment rate of 25% in soil^24,28,30,31^. Therefore, FA, being an alumino-silicate compound, can be transformed into a water sorbing material without any pretreatment. Transformation of FA into a water sorbing material can alleviate the negative effects of drought along with the nutritional enhancement of soil. However, there are not many studies that explored such a possibility.

This study demonstrated a method to synthesize an eco-friendly fly ash water absorbent (FAWA) by grafting the polyacrylic acid (PAA) on to the surface of FA in the presence of a cross-linker, N,N′-methylene-bisacrylamide. The utilization of industrial solid waste material for the synthesis of FAWA results in low production costs. The performance of the FAWA was evaluated by measuring its microstructure, WAC, swelling kinetics, re-swelling ability, sensitivity to different salts, and pH. The efficacy of the FAWA for reducing the irrigation water requirement was demonstrated by mixing it with three different textured soils at three different application rates (0.1%, 0.2%, and 0.4% on w/w basis).

## Materials and methods

The raw fly ash (FA) sample was collected from the electrostatic precipitator of National Thermal Power Corporation (NTPC) Limited, Farakka. All the basic physicochemical properties of the used FA were characterized and presented in Table S1 (in supplementary file). Acrylic acid (AA) [purity 99%], and N,N′-methylene-bisacrylamide (MBA) [purity 99.5%] was purchased from Sigma Aldrich, Bangalore, India. Ammonium persulfate (APS) [purity 98%] and sodium hydroxide (NaOH) [Purity 98%] were procured from Merck Specialties Private Limited, India. All the procured reagents were of analytical grade and used without further refinement. Distilled water was used to prepare all the stock solutions throughout this work. Three different textured natural soils were collected from different locations of the north-eastern region of India for this study. The collected soils were air-dried and sieved as per ASTM standard^32^, and only particles finer than 2 mm sieve size were considered. The basic physical properties and mineralogical composition of the selected soil samples were presented in Table S2, along with their USDA classification^33^. The soils were selected in such a manner that the soil textural influence on the performance of FAWA for drought management can be appraised.

### Synthesis of fly ash water absorbent (FAWA)

A series of FAWA samples were prepared by considering different combinations of various amounts of FA (backbone material), N,N′-methylene-bisacrylamide (MBA) (cross-linker), ammonium persulfate (APS) (initiator), and AA (monomer) with different neutralization degree (neutralized with NaOH). Every combination was repeated thrice to ensure repeatability of test results. A total of 360 number of combinations (including repetitions) were performed for the synthesis of FAWA. Out of these, the most important combinations (i.e., 24 numbers) are listed in Table S3, which was further used to optimize the reagent quantities for achieving maximum WAC. For optimizing the reagent content, only one reagent amount was varied at a time while others kept constant. It may be noted that the monomer content was kept constant at 8 g, and the polymerization reaction temperature was chosen as 70 °C throughout this study based on the literature^7,8,19,20^.

**Figure 1:**
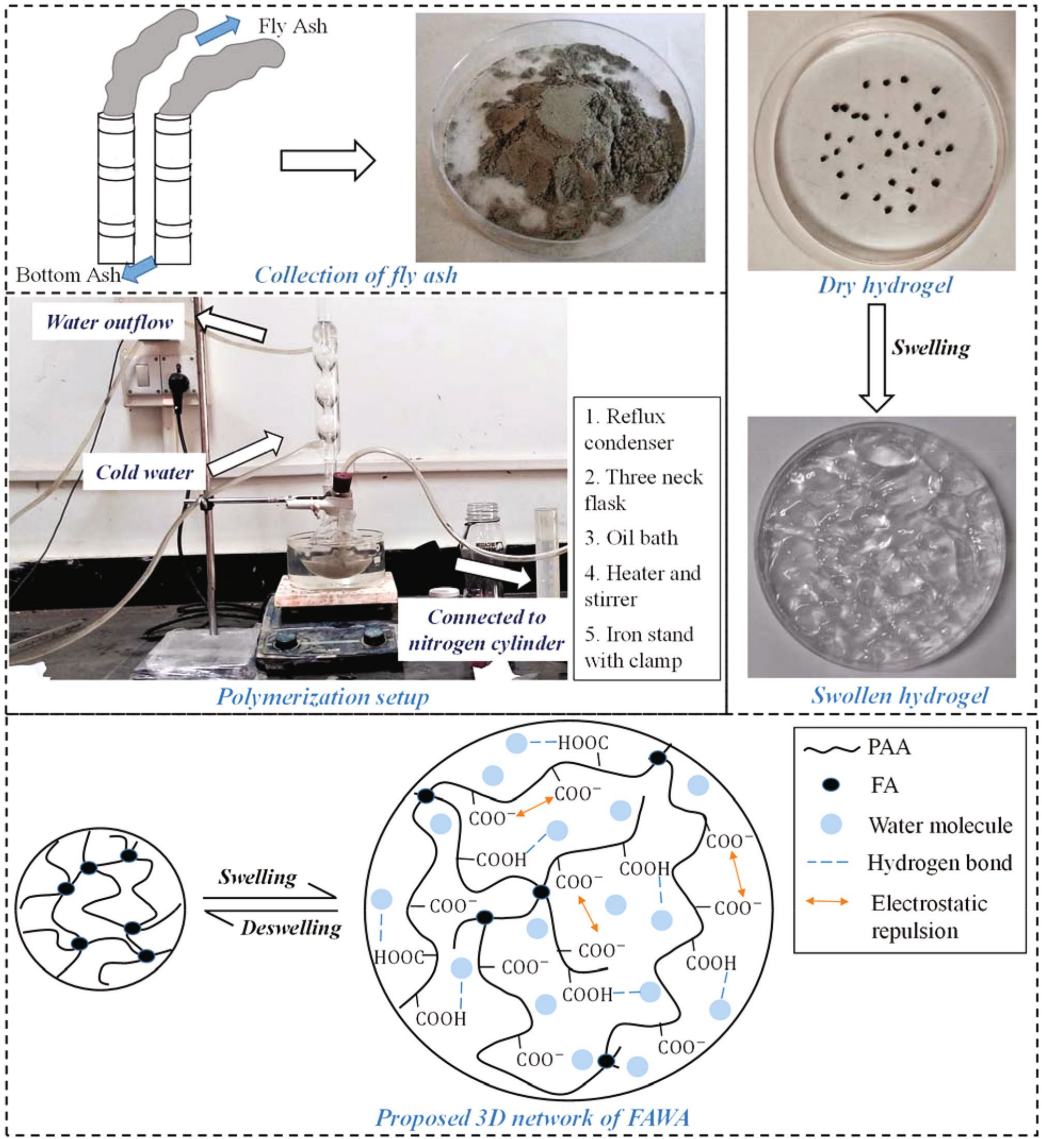
Basic overview of the FAWA synthesis process and its swelling mechanism.

**Figure 2:**
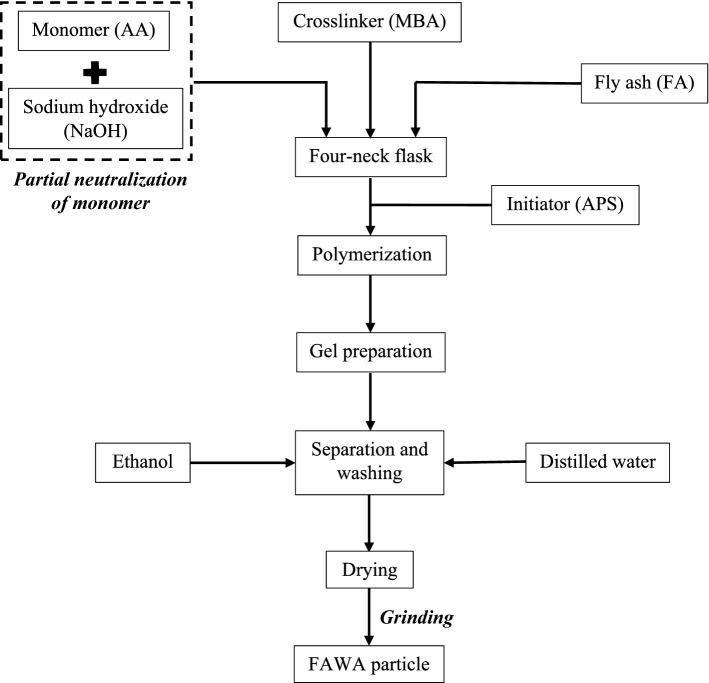
Flowchart explaining the synthesis scheme of FAWA production.

To begin with the synthesis, a certain amount of partially neutralized AA monomer was dissolved in 30 mL of distilled water in a 250 mL four neck flask equipped with a reflux condenser, thermometer, mechanical stirrer and nitrogen line, as shown in Fig. 1. FA powder was then added to the aforementioned partially neutralized monomer solution, and the flask was connected to a nitrogen cylinder for 30 min to remove the dissolved oxygen from the solvent. Thereafter, under the nitrogen environment, a certain amount of MBA, initiator APS, and distilled water were added to the mixture with effective stirring. The mixture was slowly heated in an oil bath at 70 °C for 2 h to complete the polymerization reaction. Subsequently, the resultant product was dried at 80 °C to a constant weight in an oven. The dried product was washed several times with distilled water and ethanol to remove the unreacted reagent. Finally, the obtained FAWA was dried at 80 °C and milled to particle size in the range of 10–50 mesh (0.3–2 mm). A flow chart was presented in Fig. 2 to describe the synthesis process of FAWA production. A control sample without the FA was also prepared following the same process as described above, designated as polyacrylic acid (PAA).

### Characterization of FAWA

The functional groups of FA, PAA, and FAWA were characterized using a Perkin–Elmer FTIR operated in a range from 4000 to 450 cm^−1^. For this purpose, the dried sample was mixed with dried potassium bromide (KBr, optical grade) powder and pressed into small slices for further measurement of the spectrum.

The mineralogy of the parent and final products were determined by the X-Ray Diffractometer (Company: Rigaku, Model No: TTRAX III). The selected range of 2θ was 5° to 70° with a scanning speed of 2°/min.

The surface morphology of FA, PAA, and FAWA was obtained using a field emission scanning electron microscope (FESEM) (Zeiss Sigma, Oberkochen, Germany). For FESEM analysis, the samples were mounted on aluminum stubs coated with double-sided carbon tape.

The Brunauer–Emmett–Teller (BET) surface area of FA and FAWA was determined by N_2_ adsorption–desorption isotherm. Prior to the analysis, 0.5 g of sample was degassed at 130 °C for 4 h, followed by N_2_ adsorption at 77 K.

The zeta potential and dynamic light scattering (DLS) analysis of FA and FAWA was carried out using Zetasizer Nano ZS90 (model no. ZEN3690). The samples were added to distilled water at a temperature of 25 °C prior to the measurement. Three identical samples were prepared, and the mean value, along with the standard deviation, was considered and reported below.

### Measurement of swelling characteristics

The swelling characteristics of FAWA include swelling kinetics, water-absorbing capacity (WAC), and re-swelling capability under alternate wetting–drying. These characteristics are mandatory for establishing the efficacy of FAWA for drought management.

### Swelling kinetics

Evaluation of swelling kinetics is important to understand the mechanism of the swelling process as well as the absorption rate and time to reach swelling equilibrium. To evaluate the swelling kinetics of the FAWA, 0.2 g of dry material was taken in a nylon teabag and immersed into 250 mL beaker with a sufficient amount of distilled and tap water (pH = 6.5; Electrical conductivity = 0.11 mS/cm). The teabag was lifted from the water at predetermined time intervals and drained for 2 min. Thereafter, the sample was weighed, and the water absorbency with time was calculated by Eq. (1), after deducting the weight of the teabag. A highly sensitive high precision microbalance (readability = 0.1 mg) was used for weighing the samples. The procedure was repeated until the water absorbency reached the equilibrium swelling.

1 $${Q}_{t}=\frac{{W}_{wet\left(t\right)}-{W}_{dry}}{{W}_{dry}}$$

here, ${Q}_{t}$ (g/g) is the water absorbency at time t; *W*_*wet(t)*_, and *W*_*dry*_ represent the water-swollen weight at time t, and dry weight of FAWA. The WAC was obtained from the equilibrium swelling and reported as grams of water per gram of dry FAWA. For all the cases, three samples were used to ensure repeatability of the measured data.

### Re-swelling capability

Re-swelling capability is one of the most crucial factors determining the performance of FAWA for field applications. The re-swelling ability of the FAWA was investigated through multiple alternate wetting–drying cycles and measuring its WAC. For this purpose, a weighed amount of FAWA was added in 250 mL of the swelling medium at ambient temperature for 4 h to achieve complete saturation (based on swelling results). The swollen FAWA was filtered, and WAC was measured following a similar procedure, as discussed above. The swollen material was then dried in an oven at 80 °C until a constant weight was reached. Thereafter, the dried sample was again added in 250 mL of swelling medium for the next cycle of WAC measurement. The procedure was repeated for eight cycles of wetting–drying to obtain the re-swelling ability of the FAWA. These results are needed to assess the deterioration of FAWA in the field with time due to seasonal effects.

### Salt sensitivity

To investigate the effect of salt concentration, a known quantity of dry FAWA was added to different concentrations of salt solution and allowed to swell until the equilibrium is reached. The WAC at the particular salt concentrations was calculated from Eq. (1). The WAC of FAWA was evaluated in seven different inorganic salts including sodium chloride, potassium chloride, ammonium chloride, calcium chloride, sodium nitrate, sodium carbonate, and sodium sulfate at six different molar concentrations (0.01 M, 0.05 M, 0.1 M, 0.15 M, 0.2 M, and 0.3 M). The choice of the salts facilitated to compare the sensitivity of FAWA to various cations (${\mathrm{Na}}^{+}$, ${\mathrm{K}}^{+}$, ${\mathrm{Ca}}^{2+}$, ${\mathrm{NH}}_{4}^{+}$) and anions (${\mathrm{Cl}}^{-}$, ${\mathrm{NO}}_{3}^{-}$,${\mathrm{CO}}_{3}^{2-}$, ${\mathrm{SO}}_{4}^{2-}$) specifically.

### pH sensitivity

To verify the influence of pH, four different stock solutions, including HCl, H_2_SO_4_, NaOH, and CaO, having pH ranging between 2.0 and 12.0, were used. No additional ions were added through the buffer solution for setting the pH as the WAC is strongly affected by the ionic strength of the solution. The stock solutions were diluted with distilled water to reach the desired acidic and basic pH values. The main reason for using different valence stock solutions was to critically evaluate any effect of the composition of the stock solutions on WAC of FAWA. In each of the cases, WAC for a particular pH was calculated following the method discussed earlier.

### Drying characteristics and evaporation rate of FAWA amended soil

The FAWA was mixed with three different textured soils (including Sand, Silt loam, and Clay loam) at three different application rates of 0.1%, 0.2%, and 0.4% (w/w). This range of FAWA application rate was selected based on the previous literature^34–36^. The air-dried soil samples (50 g each) were mixed with the required amount of dry FAWA and placed in a plastic container (diameter = 4.5 cm) with perforated bottom fitted with filter paper. Each plastic container was immersed in distilled water for 24 h to allow full saturation. Thereafter, the gravimetric water content (GWC) of the soil-FAWA mix was calculated using the Eq. (2). Each of the containers was kept in ambient condition (Avg. temp = 25 °C and Avg. RH = 70%) for drying, and the weight of the containers was measured after every 24 h to obtain the drying characteristics of the FAWA amended soil. Further, the evaporation rate (ER) for each sample was calculated using Eq. (3). A total of 36 sets of the sample were prepared for this experiment, including three repetitions for each concentration of FAWA.

2 $$GWC=\frac{weight \,of \,absorbed \,water}{dry \,weight \,of \,soil+FAWA}$$

3 $$ER (mm/day)=\frac{Amount \,of \,water \,evaporated \,per \,day \,(m{m}^{3})}{Cross \,sectional \,area \,of \,container \,(m{m}^{2})}$$

## Results and discussion

### Synthesis and characterization of FAWA

The FAWA is formed by graft polymerization of acrylic acid (AA) monomer on the surface of FA in the presence of cross-linker MBA and radical initiator APS. In the presence of FA, a large amount of AA monomer could be captured on the surface of FA due to hydrogen-bond interaction between the functional group present in FA and AA molecule. During heating at 70 °C, the initiator (i.e., APS) in the solution was decomposed to generate sulfate anion radicals (SAR). Subsequently, SAR initiates the polymerization process by activating the monomer (AA). These active monomer radicals act as a free radical donor to the adjacent monomers, and thus, the propagation of the homo-polymer started. The SAR also creates a chemically active group in the FA, which acts as an active center for the chain propagation. The polymer chain was captured on these active FA centers, leading to the growth of the grafted polymeric chain. During the polymerization reaction, these grafted polymer chains react with the end vinyl group present in the cross-linker, MBA, to produce an interpenetrating, three-dimensional (3D) polymeric network with a lot of free carboxyl groups. In this way, the FA particle plays two significant roles in the formation of FAWA. Firstly, the functional group present in FA captures AA monomer and cross-linker MBA, resulting in the graft polymerization reaction. Secondly, the FA particle in the polymer network acts as an additional network point, which enhances the mechanical stability and salt resistivity of the water-absorbent.

**Figure 3:**
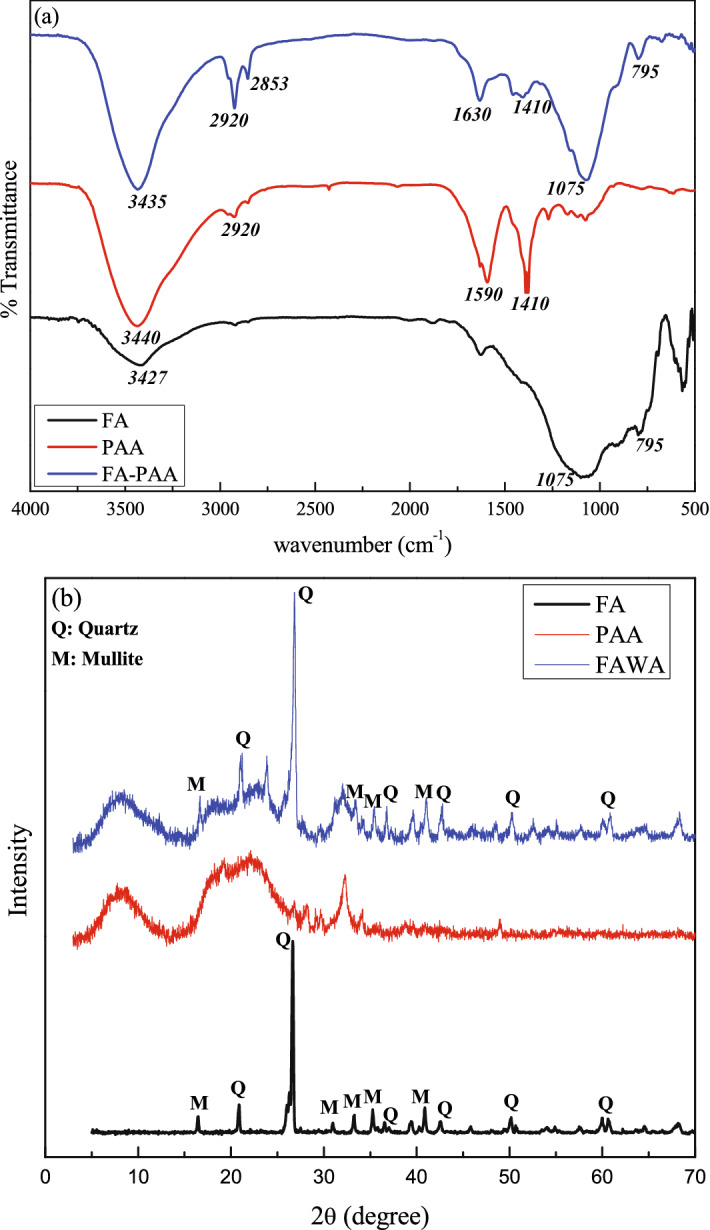
(**a**) FTIR spectrum and (**b**) XRD analysis of FA, PAA and FAWA.

The FTIR spectroscopy was used to characterize the functional groups of parent material and the final product. The FTIR spectrum of the FA, PAA, and FAWA are presented in Fig. 3a. As observed from the figure, the broad peak in FA observed at 3427 cm^−1^ is due to the existence of H-bonded (i.e., intermolecular and intramolecular) hydroxyl stretching vibration. The peaks observed at 1088 cm^−1^ and 795 cm^−1^ were assigned to asymmetrical stretching of Si–O–Si and symmetrical stretching of Si–O–Si, which can also be seen in the FAWA. In addition to this, the new absorption peaks in the region of 2920 cm^−1^ and 2853 cm^–1^ in FAWA were ascribed to the stretching of the alkyl C–H bond. The characteristic IR signature at 1410 cm^−1^ and 1630 cm^−1^ for symmetric and asymmetric stretching of carboxylate groups (COO^‒^) validating the successful grafting of AA on the surface of FA. Overall, the FTIR spectrum of synthesized FAWA indicated the existence of both PAA and FA.

The XRD spectrum of FA, PAA, and FAWA are presented in Fig. 3b. The XRD pattern of FA clearly indicates that it consists of quartz (Q) and mullite (M). The XRD pattern of PAA exhibit a broad peak at 2θ = 22°, which indicates an amorphous structure with low crystallinity. A Similar peak for PAA was reported in the previous literature^37,38^. The presence of quartz and mullite in FAWA, along with the same crystalline peak of PAA, confirmed the incorporation of FA in the PAA chain network.

**Figure 4:**
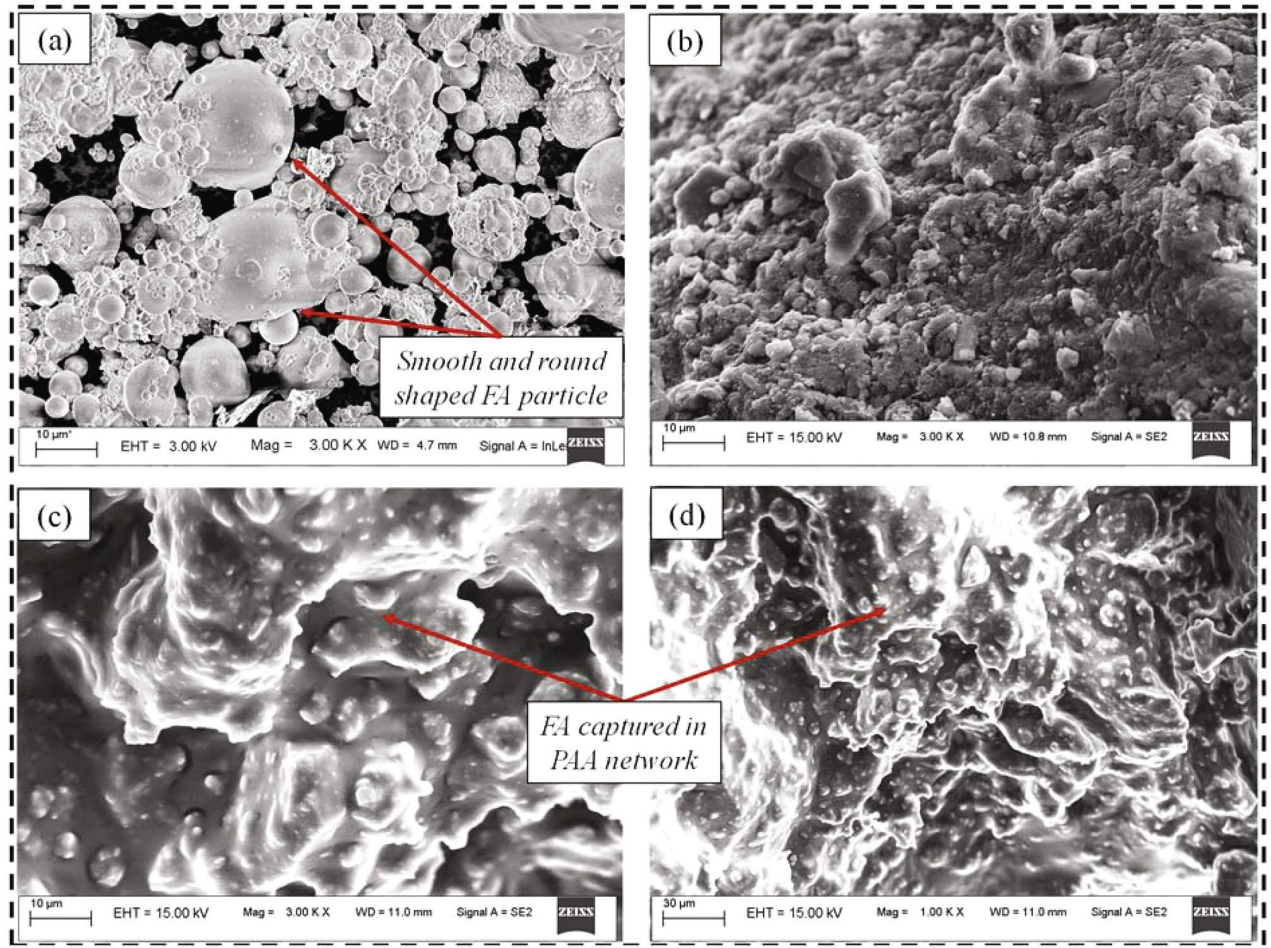
FESEM image of (**a**) raw FA, (**b**) PAA, (**c**) FAWA at 3000× and (**d**) FAWA at 1000× magnification.

The graft polymerization of PAA on the surface of FA can be further verified through their surface morphology. For this purpose, the surface morphology of the FA, PAA, and FAWA, as visualized from FESEM, presented in Fig. 4. Figure 4a depicts the FA particles, which are relatively smooth and spherically shaped. On the other hand, the morphology of the PAA displayed a compact, flat, and porous surface (Fig. 4b). The surface profile of FAWA shown in Fig. 4c,d are distinctly different from PAA. The FAWA portrayed a comparatively coarse, loose, and porous surface with a considerable number of cavities, indicating more water-absorbing sites as compared to PAA. The change in the surface morphology was due to the grafting of FA, which destroyed the tight, smooth surface of the PAA, leading to a heterogeneous and loose structure with a lot of cavities.

The adsorption–desorption isotherm and pore size distribution of FA and FAWA were presented in Fig. S4, along with the BET surface area and average pore diameter in Table S5. It can be observed that the surface area decreased from 2.07 m^2^/g (for FA) to 0.08 m^2^/g in FAWA. This can be attributed to the pore-blocking effect because significant pores in FAWA is occupied by sodium polyacrylate. A similar pore-blocking effect was also reported in He et al.^39^. Moreover, such a low surface area of FAWA suggests that the water absorption mechanism is not primarily governed by surface properties.

The zeta potential (reflects the surface charge of the material) of FA and FAWA were found to be − 3.44 ± 0.18 mV, and − 4.82 ± 0.76 mV. The increase in zeta potential in FAWA can be attributed to the existence of carboxylate groups (COO^‒^) on the surface after modification.

### Optimizing the synthesis of FAWA for maximum WAC

**Figure 5:**
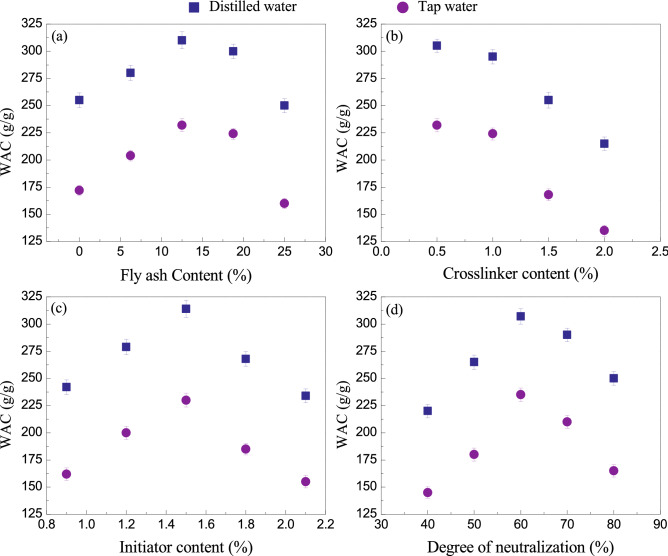
Effect of (**a**) FA content, (**b**) cross-linker content (**c**) initiator content and (**d**) neutralization degree of AA on WAC of the synthesized FAWA.

The maximum WAC of FAWA is governed by the optimal content of parent materials used in graft polymerization, which include FA content, cross-linker content (MBA), initiator content (APS), neutralization degree of AA, and water dilution. The influence of these parameters on the WAC was evaluated and presented in Fig. 5. The variation in FA content, cross-linker content, and initiator content was expressed as a weight percentage with respect to the dry weight of monomer AA. The effect of different amounts of FA content on the WAC in distilled water and tap water can be observed from Fig. 5a. With an increase in FA content from 0 to 25%, the WAC of FAWA increased to 310 g/g and then decreased to 255 g/g in distilled water. A small quantity of FA act as an additional network point and react with the monomer, which enhances the three-dimensional polymeric network due to which the water absorbency increases. A further increase in FA content increases the density of crosslinking in the polymer network. It is therefore invariably necessary to identify an optimal FA content (= 12.5% according to this study) for WAC of FAWA. The quantity of FA proposed for the synthesis of FAWA is well below the recommended maximum value of FA application rate in agriculture (= 25%)^30^.

Figure 5b shows the effect of cross-linker content on the WAC. The figure depicts that the WAC is inversely proportional to the cross-linker content. With the increase in cross-linker content, the crosslinking density of the polymer increases, leading to a much tighter network with fewer free nodes in the polymer chain. It can be noted that cross-linker content less than 0.5% resulted in a water-soluble polymer composite, and its WAC cannot be evaluated. Therefore, the cross-linker content was fixed at 0.5%.

The effect of initiator content on the WAC is presented in Fig. 5c. The WAC increases with an increase in initiator content from 0.8% to 1.5% and then decreases with a further increase in the initiator. The purpose of the initiator in the polymerization reaction is to produce free radical sites on FA particle and AA monomer so that the monomer could be grafted well on the surface of FA. Hence, the WAC increases with the initial increase in initiator content. However, exceeding the initiator content beyond 1.5% lead to an increased number of radical active sites in the monomer, and chain propagation gets terminated^18,40,41^. This results in a decrease in the molecular weight of the polymer chain, and subsequently, the WAC is reduced.

Figure 5d shows that the neutralization degree of AA has a significant effect on the WAC of the FAWA. The optimum WAC of 310 g/g in distilled water and 230 g/g in tap water was obtained at 60% neutralization degree of AA. Neutralization of AA with sodium hydroxide (NaOH) leads to an increase in the strong hydrophilic group (–COONa) in the polymer network. The negatively charged carboxyl groups (–COO^−^) creates an anion–anion electronic repulsion within their polymeric network. On contact with water, a significant pressure difference is developed between the polymer network and water, which supports the penetration of water molecules into the polymer network. After exceeding the optimum value of the neutralization degree, the WAC decreases because of the increase in Na^+^ counter ions, which shields the negatively charged carboxyl group.

To evaluate the effect of water, the polymerization reaction was performed by adding 20 mL, 30 mL, 40 mL, 50 mL, and 60 mL of distilled water into the reaction mixture. It was observed that the polymerization did not take place when the amount of water was lower than 30 mL and higher than 60 mL. The WAC of synthesized FAWA remained constant (= 310 g/g) for the amount of water ranging between 30 and 60 mL. It can be noted that the WAC of the synthesized FAWA is comparable with the WAC of other laboratory grade and commercial grade SAH^42–48^ (Table 2).

**Table 2:** Comparison of WAC of FAWA with other available SAH.

Reference	WAC in distilled water (g/g)	Composition
Wan et al.^42^	430	Polyacrylic acid, acrylamide, kaolinite
Rodrigues et al.^43^	225	Polyacrylic acid, chitosan, rice husk
Wan et al.^44^	360	Polyacrylic acid, acrylamide, corn stalk
Xu et al.^45^	252	Polyacrylic acid, sea buckthorn
Xiao et al.^46^	253	Polyacrylic acid, acrylamide, corn starch
Abdallah^47^	244	Polyacrylamide (commercial SAH)
Saha et al.^48^	280	Polyacrylic acid (commercial SAH)
Present study	310	Polyacrylic acid, fly ash

### Swelling kinetics of FAWA

**Figure 6:**
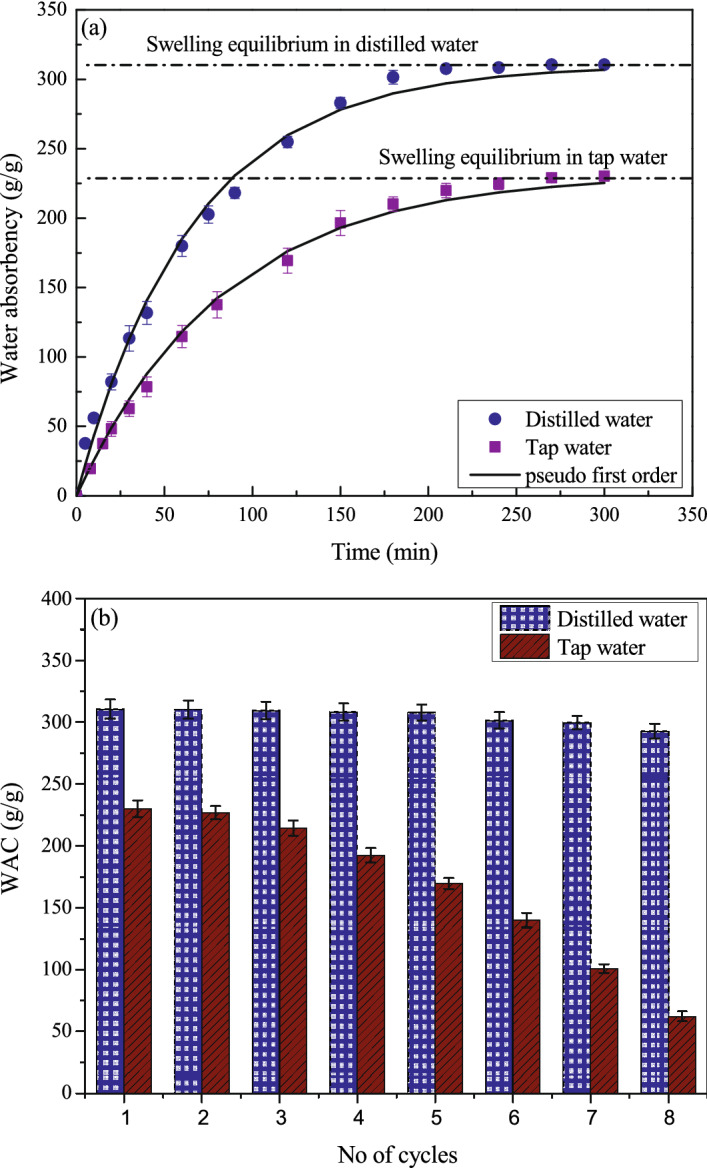
(**a**) Swelling kinetic and (**b**) reswelling ability of FAWA in distilled and tap water.

The swelling kinetics of FAWA in distilled and tap water is presented in Fig. 6a. The swelling trend in both solutions is found to be similar. The swelling capacity increased rapidly in the initial stage and then attained a constant equilibrium swelling capacity, which is similar to the trends reported in the previous literature^41,44,49^. It can be noted that FAWA reaches its swelling equilibrium within 4 h. from the beginning of the test in both the solvent. A pseudo-first-order kinetic model (Eq. 4) was fitted to the experimental data to describe the swelling mechanism.

4 $${Q}_{t}={Q}_{e}[1-\mathrm{exp}\left({-k}_{1}t\right)]$$

here, ${Q}_{e}$ (g/g) is the equilibrium water absorbency, t is the time (min), and ${k}_{1}$ denotes the first-order rate constant (min^−1^). The value of ${k}_{1}$ was calculated from the measured swelling kinetics data using the non-linear curve fitting technique. The swelling rate constant in distilled water and tap water was found to be comparable and equal to 0.0152 min^−1^ and 0.0119 min^−1^, respectively.

### Re-swelling capability of FAWA

Figure 6b represents the re-swelling capabilities of FAWA in distilled and tap water as a function of alternate wetting–drying cycles (8 cycles). A negligible decrease in the WAC was noticed after eight wetting–drying cycles for FAWA in distilled water, whereas a sharp decrease in the WAC can be observed in tap water. The minimal decrease in WAC associated with distilled water may be due to change in the polymeric network as the FAWA was dried in the oven. However, the sharp decrease in the WAC in tap water could be a result of the presence of salt and other impurities in tap water, which affected the polymer chain and weakened the chemical bond between different hydrophilic groups, leading to degradation of the polymeric structure. The decrement in WAC for FAWA in tap water was found to be 73% after eight wetting–drying cycles. In contrast, the WAC decrement was only about 6% in the case of distilled water. These results indicate that the FAWA has an excellent re-swelling ability and can efficiently contribute towards water retention during drought stress even after the eighth alternate drying–wetting cycle. The quality of pore water has a significant influence on the re-swelling capability of FAWA. This opens up the need for further research for deciding the time interval for replenishing FAWA in the field based on soil quality.

### Salt sensitivity of FAWA

**Figure 7:**
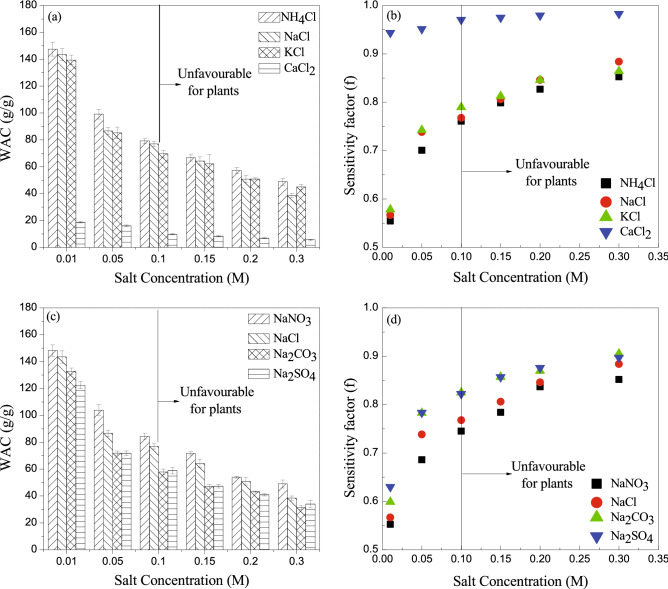
Sensitivity of FAWA to different salt solutions.

The effect of various inorganic salt ions on the WAC of the FAWA was investigated and presented in Fig. 7. It can be observed that the WAC was significantly decreased with the increase in the salinity level. A rapid decrease in the absorbency can be noticed up to a salt concentration of 0.05 M, followed by a minimal decrement beyond 0.05 M. This was attributed to the increase in ionic strength of the salt solution and subsequent reduction in the osmotic pressure difference between the solvent and polymer network. It was also observed that the presence of divalent ions affected the WAC more than monovalent ions. This was due to the higher ionic strength of divalent ionic salt compared to the monovalent ionic salt. According to Eq. (5) suggested by Hermans^50^, the swelling characteristics of any SAH is significantly influenced by the ionic strength of the solution.

5 $${Q}^\frac{5}{3}=A+B\frac{{i}^{2}}{I}$$

here, *Q* denotes the WAC, *i* denotes the concentration of the charges bound to the gel, *I* denote the ionic strength of the solvent and *A*, *B* are empirical parameters.

Figure 7a shows the effect of different monovalent (${\mathrm{Na}}^{+}$, ${\mathrm{K}}^{+}$, ${\mathrm{NH}}_{4}^{+}$) and divalent (${\mathrm{Ca}}^{2+}$) cations on WAC of FAWA in the presence of a common anion (${\mathrm{Cl}}^{-}$). The order of WAC of FAWA in the chloride salt solution was found to be NH_4_Cl > NaCl > KCl > CaCl_2_. Out of the three monovalent cations, the effect of ${\mathrm{NH}}_{4}^{+}$ ions on WAC of FAWA was found less effective as the other two cations are more electro-positive since they belong to S-block elements in the periodic table. The FAWA showed higher WAC in NaCl solution compared to KCl as the size of the Na^+^ ion is small compared to the K^+^ ion. On the other hand, the effect of divalent Ca^2+^ ion on WAC was found to be much higher in comparison to the monovalent ions because of the higher ionic strength. In addition, Ca^2+^ ion can increase the crosslinking density of the polymer leading to a reduction in free available water-absorbing sites within the polymer network^17^.

The effect of monovalent (${\mathrm{Cl}}^{-}$, ${\mathrm{NO}}_{3}^{-}$) and divalent (${\mathrm{CO}}_{3}^{2-}$, ${\mathrm{SO}}_{4}^{2-}$) anions on WAC in the presence of a common cation (${\mathrm{Na}}^{+}$) are presented in Fig. 7c. The decreasing order of WAC of the polymer in different sodium salts were found to be NaNO_3_ > NaCl > Na_2_CO_3_ > Na_2_SO_4_. It is obvious that the WAC was found to be more in nitrate (${\mathrm{NO}}_{3}^{-}$) and chloride (${\mathrm{Cl}}^{-}$) compared to carbonate (${\mathrm{CO}}_{3}^{2-}$) and sulfate (${\mathrm{SO}}_{4}^{2-}$) ions as the former ions are monovalent. Among the monovalent anions, the effect of ${\mathrm{Cl}}^{-}$ on WAC was more than ${\mathrm{NO}}_{3}^{-}$ ions. This was attributed to the fact that NaCl is formed from a more acidic group [HCl (pK_a_ = − 7)], whereas NaNO_3_ is formed from a less acidic group [HNO_3_ (pK_a_ = − 1.3)]. Due to the same reason, among the divalent anions, the effect of Na_2_CO_3_ salt is lesser than Na_2_SO_4_ salt on the WAC of FAWA.

Soil salinity is a condition characterized by a high concentration of soluble salts, of which NaCl is the most soluble and widespread salt^51^. Although soil salinity is a complex phenomenon resulted from different salt sources, irrigation combined with poor drainage is the principal source adding calcium (Ca^2+^), magnesium (Mg^2+^), and sodium (Na^+^) to soil^52^. As a result of water evaporation, Ca^2+^ and Mg^2+^ often precipitate into carbonates, leaving Na^+^ dominant in the soil^53^, and therefore, Na^+^ concentrations often exceed those of most macronutrients by one or two orders of magnitude, and by even more in the case of micronutrients. Increases in cations and their salts, NaCl in particular, in the soil generates external osmotic potential, which prevents or reduces the water influx into the root, resulting in water deficit similar to drought conditions^54^. The use of water-absorbent like FAWA can improve the water availability to the plant roots as compared to the bare soil.

For a comparative measure of the sensitivity of FAWA to a particular type of aqueous fluid, a dimensionless salt sensitivity factor (*f*) was calculated from Eq. (6). The calculated f value for the used salts at different molar concentrations was presented in Fig. 7b,d. As expected, the f value of FAWA in divalent ions was much higher than the monovalent ions.

6 $$f=1- \frac{WAC\, in \,salt \,solution}{WAC \,in \,distilled \,water}$$

Though the effect of salt ions on the WAC of FAWA was investigated up to 0.3 M, the crop species can only sustain up to a soil salinity level of 0.1 M^55,56^. Beyond this salinity level, the plants start to wilt due to ion toxicity, and the plant growth is completely prevented by the salt ions^57^. It can be observed that the synthesized FAWA has WAC of 80 g/g even up to 0.1 M salinity level for monovalent ions, which showed its excellent potential for agricultural application under water stress conditions.

### pH sensitivity of FAWA

**Figure 8:**
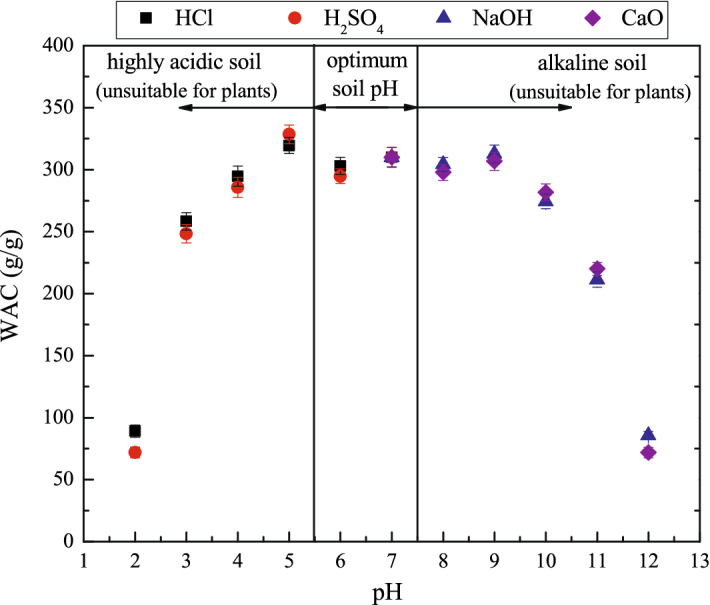
Effect of pH on the WAC of FAWA.

The sensitivity of FAWA in terms of WAC to various pH solutions was presented in Fig. 8. A significant variation in the WAC can be observed in FAWA at a wide range of pH due to the presence of different interacting species in the swelling medium. The influence of different stock solutions on the WAC was found very minimal at the same pH value. Various phenomenon and mechanisms are involved during the swelling of FAWA at different pH range. At very low pH (pH < 3.0), the main interacting species present in the solution are protonated ($-{\mathrm{COOH}}_{2}$), and excess acid anions (${\mathrm{Cl}}^{-}$, ${\mathrm{SO}}_{4}^{2-}$). Presence of these excess anions shield the charge of protonated carboxyl cation, which prevent the electrostatic repulsion between protonated ($-{\mathrm{COOH}}_{2}$), resulting in a remarkable decrease in WAC. At pH range 5.0–6.0, an intense repulsion between protonated ($-{\mathrm{COOH}}_{2}$) cause a significant increase in osmotic pressure inside the FAWA. This high osmotic pressure difference between the polymer network and the external solution is balanced by the swelling of the FAWA particles. At neutral pH, the majority of base and acid groups are in non-ionized form. As a result, the interchain hydrogen bond form between some of the carboxyl groups (–COOH) of the monomer leading to some minor decrease in the WAC. Similarly, for the pH range 8.0–10.0, electrostatic repulsion between multiple deprotonated ($-{\mathrm{COO}}^{-}$) increase the charge density inside the particle resulting in high swelling in the solution. With the increase in pH (pH > 10.0), the presence of different basic cation like Na^+^, Ca^2+^ increases, which shield the charge of deprotonated ($-{\mathrm{COO}}^{-}$) that causes a significant decrease in the WAC. A similar type of observation was reported in Mahdavinia et al.^58^. According to USDA (United States Department of Agriculture) Natural Resources Conservation Service^59^, soil pH can vary from 5.5–8.5 with extreme scenarios of 3.5–9.0. It was reported that the optimum soil pH range for most of the plants is 5.5–7.5, beyond which the plants are susceptible to aluminum toxicity (for pH less than 5.5) and nutrient deficiency (for pH higher than 7.5)^60^. From Fig. 8, it is evident that the WAC of the synthesized FAWA is negligibly affected by soil pH.

### Drying characteristic and evaporation rate of FAWA amended soil

**Figure 9:**
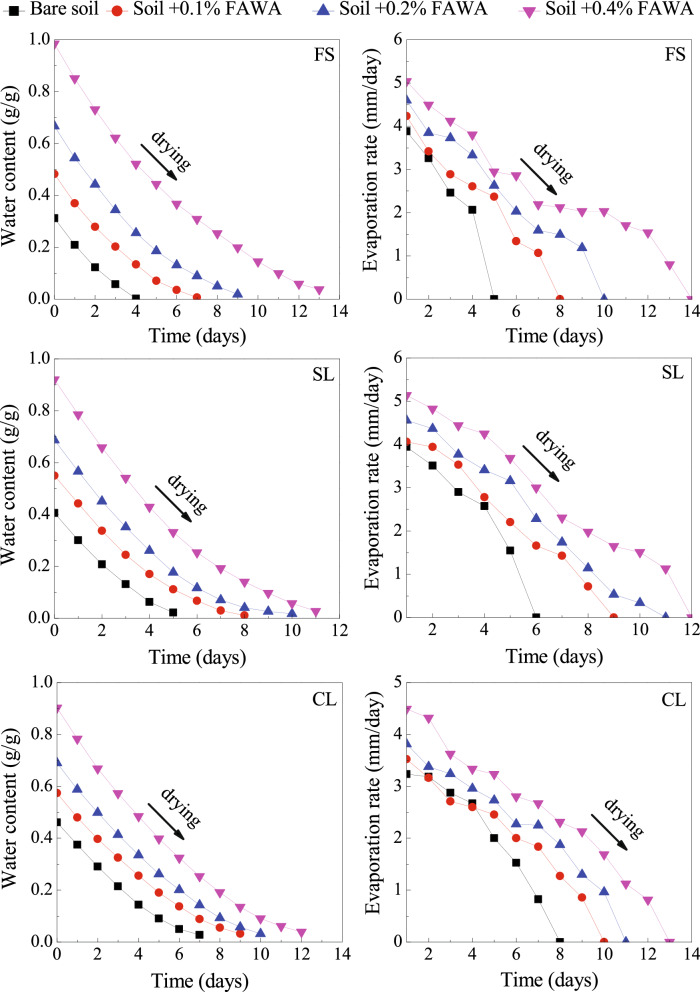
Drying characteristics of the FAWA amended soils.

The performance of the synthesized FAWA was evaluated by measuring the drying characteristic of the amended soils in terms of gravimetric water content (w) as a function of time. The drying characteristics of FAWA amended soils were depicted in Fig. 9. It can be observed that the addition of FAWA has increased the desaturation time (i.e., time taken for complete water loss) for all the soils. With the 0.4% FAWA amendment, the desaturation time increased by a factor of 3.3, 2.2, and 1.5 in FS, SL, and CL, respectively. This is due to the increase in soil–water storage with the FAWA addition. The increase in soil–water storage is associated with the maximum water content (*w*_*max*_) of soil (at time t = 0), which increases with the FAWA concentration, irrespective of soil texture. At the highest application rate (0.4%), the *w*_*max*_ value of FS was increased by 3.3 times as compared to bare soil, whereas for the SL and CL, the increment was 2.3 times and 1.9 times, respectively. The higher improvement of *w*_*max*_ value for coarse-textured soil (FS) can be attributed to a relatively larger size of soil pore diameter as compared to fine-textured soil (SL, CL). Due to this reason, the FAWA can easily swell to its maximum swelling capacity in coarse-textured soils^48^. On the other hand, the swelling of the FAWA is restricted by the surrounding soil particle in fine-textured soil. To prove this point, the specific amount of water absorbed (SWA) by a unit mass of FAWA was evaluated by Eq. (7).

7 $$SWA (g/g) =\frac{m-b-h}{h}$$

here, m and b are the wet weight of FAWA amended soil and bare soil, respectively at t = 0; h is the dry weight of the FAWA at t = 0. The SWA value for FAWA in three different soil texture is presented in Table 3. It is quite evident that the amount of absorbed water (or swelling) by FAWA is more in FS (coarse-textured soil) as compared to SL and CL (fine-textured soil) due to larger pore geometry.

**Table 3:** SWA per unit weight of FAWA in the used soils.

Soil type		SWA (g/g)
FS	Soil + 0.1% WAP	210
	Soil + 0.2% WAP	204
	Soil + 0.4% WAP	200
SL	Soil + 0.1% WAP	177
	Soil + 0.2% WAP	174
	Soil + 0.4% WAP	168
CL	Soil + 0.1% WAP	136
	Soil + 0.2% WAP	134
	Soil + 0.4% WAP	130

To understand the influence of FAWA addition on drying rate of soil, the evaporation rate was calculated by Eq. (3), and presented in Fig. 9. The rate of evaporation was found to be higher in FAWA amended soils as compared to bare soils, and the evaporation rate increases with the FAWA concentration. This gives an indication that the absorbed water gets released easily in the FAWA amended soils, suggesting plant roots can easily extract water from the amended soil. Based on these results, it is quite evident that the application of the synthesized FAWA can effectively maximize the irrigation interval (time gap between two successive irrigation), and increase the availability of water to the plant roots. The application rate of FAWA used in this study is much less as compared to the recommended amount of FA application in the agricultural field (i.e., 25%) proposed in the literature^30,31^. Therefore, the environmental impact of FAWA application in soils is negligible. It may be noted that the reported application rates of FAWA (0.1%, 0.2%, and 0.4%) are for pot experiments. Further studies are required to obtain the optimum application rate of FAWA for field conditions, considering the soil–water–plant–atmosphere interaction.

## Conclusions

A novel and eco-friendly fly ash water absorbent (FAWA) was synthesized by grafting the partially neutralized polyacrylic acid (PAA) chain on to the surface of fly ash (FA) using graft polymerization technique. The FTIR, FESEM, and XRD results confirm the successful grafting of PAA on the FA surface. The optimization of various parent materials for FAWA synthesis (includes FA content, cross-linker content, initiator content and neutralization degree of monomer) resulted in a water-absorbing capacity (WAC) of 310 g/g in distilled water, and 230 g/g in tap water, which is comparable with commercially available superabsorbent hydrogel (SAH). The swelling kinetics of FAWA showed that the equilibrium swelling capacity was achieved within 4 h and exhibited excellent re-swelling ability that is ideal for agricultural applications. The WAC of the FAWA was significantly affected by the salinity and pH of the external solution. The FAWA is more sensitive to multivalent ions as compared to monovalent ions due to the higher ionic strength of the former. The order of sensitivity of the FAWA for various cations was found to be in the order of ${\mathrm{NH}}_{4}^{+}$<${\mathrm{Na}}^{+}$<${\mathrm{K}}^{+}$<${\mathrm{Ca}}^{2+}$, while for anions it was found to be ${\mathrm{NO}}_{3}^{-}$<${\mathrm{Cl}}^{-}$<${\mathrm{CO}}_{3}^{2-}$<${\mathrm{SO}}_{4}^{2-}$. However, the FAWA showed WAC of 80 g/g at a salt concentration of 0.1 M, which is the limiting value for plant survival under ion toxicity. Hence, the application of FAWA will be beneficial under the saline condition as compared to bare soil. The effect of solution pH on the WAC was negligible for the pH range of 5.5 to 7.5, which is the recommended pH range of soil for plant growth. The drying characteristics of the amended soil showed that the 0.4% FAWA addition had increased the desaturation time by a factor of 3.3, 2.2 and 1.5 in fine sand (FS), silt loam (SL), and clay loam (CL), respectively. This increase in the desaturation time is associated with high water storage of FAWA indicated by the maximum water content (w_max_) of amended soil. The higher improvement in sandy soil was attributed to the higher available pore space that allows the FAWA particle to reach its maximum swelling. Based on the experimental results, it can be concluded that the synthesized FAWA can maximize the irrigation interval and save a considerable amount of water during water stress conditions. The advantage of this study is that the raw material, FA is a freely available waste product, while the other raw materials involved in the synthesis process are economical and easily available. Therefore, the synthesized FAWA is an economical value addition of waste FA, which is highly advantageous to agriculture, especially in drought-prone areas. Further studies are needed to evaluate the actual soil–water–plant–atmosphere interaction of FAWA amended soils through long-term, real-time monitoring under controlled and in-situ conditions.

## Data availability

The datasets generated during and/or analyzed during the current study are available from the corresponding author on reasonable request.

## Supplementary information

Supplementary Information
