# Supplementary material for: Transformation of non-water sorbing fly ash to a water sorbing material for drought management
Source: Sci Rep. 2020 Oct 29;10:18664. doi: 10.1038/s41598-020-75674-6 (PMC7596501; doi:10.1038/s41598-020-75674-6)
Supplement: Supplementary file 1 — Supplementary Information [file 41598_2020_75674_MOESM1_ESM.docx]

Supplementary information

**Transformation of Non-water Sorbing Fly Ash to a Water Sorbing Material: Synthesis and Characterization for Drought Management**

Abhisekh Saha^1^, Sreedeep Sekharan^2^, Uttam Manna^3^, and Lingaraj Sahoo^4^

^1^Research Scholar, Department of Civil Engineering, Indian Institute of Technology, Guwahati, Assam, India. Email: [abhisekh@iitg.ac.in](mailto:abhisekh@iitg.ac.in)

^2^Professor, Department of Civil Engineering, Indian Institute of Technology, Guwahati, Assam, India. Email: [srees@iitg.ac.in](mailto:srees@iitg.ac.in)

^3^Associate Professor, Department of Chemistry and Centre for Nanotechnology, Indian Institute of Technology, Guwahati, Assam, India. Email: [umanna@iitg.ac.in](mailto:umanna@iitg.ac.in)

^4^Professor, Department of Biosciences and Bioengineering, Indian Institute of Technology, Guwahati, Assam, India. Email: [ls@iitg.ac.in](mailto:umanna@iitg.ac.in)

Table S1. Basic physical and chemical properties of FA and FAWA

| Properties | | FA | FAWA |
| --- | --- | --- | --- |
| Specific Gravity | | 2.2 | 1.4 |
| pH | | 9.4 | 7.5 |
| Particle size range | | 0.002 mm-0.425 mm | 0.3 mm-2 mm |
| Plasticity | | Non-plastic | NA |
| ASTM C618 classification | | Class F | NA |
| Water absorbing capacity (g/g) [in distilled water] | | 0.4 | 310 |
| EDX analysis (elemental composition) (% wt.) | O | 53.3 | 31.1 |
|  | Si | 24.1 | 8.9 |
|  | Al | 14.1 | 4.6 |
|  | Fe | 3.6 | 0.5 |
|  | K | 1.3 | 0.2 |
|  | Ca | 1.2 | 0.3 |
|  | Mg | 0.4 | 0.2 |
|  | P | 0.2 | 0.1 |
|  | Mn | 0.2 | 0.1 |
|  | Na | - | 4.9 |
|  | C | - | 47.2 |

*NA: Not applicable

Table S2. Basic physical properties of the soils

| **Physical Properties** | | **Soil Material** | | |
| --- | --- | --- | --- | --- |
|  |  | **Fine Sand** | **Silt loam** | **Clay loam** |
| Designation | | FS | SL | CL |
| Specific Gravity (G) | | 2.65 | 2.73 | 2.69 |
| Particle Size Distribution (%) | Gravel (> 4.75 mm) | 0 | 0 | 0 |
|  | Coarse sand (2 mm – 4.75 mm) | 14  69.65  15.45  1.32  0.00 | 0 | 0 |
|  | Medium sand (0.425 mm – 2 mm) | 40 | 0 | 6 |
|  | Fine sand (0.075 mm – 0.425 mm) | 46 | 32 | 19 |
|  | Silt (0.002 mm – 0.075 mm) | 0 | 58 | 47 |
|  | Clay (< 0.002mm) | 0 | 10 | 28 |
| USDA Classification | | Sand | Silt Loam | Clay Loam |
| Free swell index (%) | | NA | NA | 12 |
| Minerals present | | Quartz | Quartz, Calcite | Quartz, Hematite |
| *NA = Not Applicable/Negligible | | | | |

Table S3. Important combinations of reagent quantity for the synthesis of FAWA

| Trial No. | Monomer  (AA) | Cross-linker  (MBA) | | FA | | Initiator  (APS) | | Neutralization degree  (%) | Water |
| --- | --- | --- | --- | --- | --- | --- | --- | --- | --- |
|  | (g) | (mg) | (%) | (g) | (%) | (mg) | (%) |  | (g) |
| Optimization of FA content | | | | | | | | | |
| 1 | 8 | 40 | 0.5 | 0 | 0 | 120 | 1.5 | 60 | 50 |
| 2 | 8 | 40 | 0.5 | 0.5 | 6.3 | 120 | 1.5 | 60 | 50 |
| 3 | 8 | 40 | 0.5 | 1 | 12.5 | 120 | 1.5 | 60 | 50 |
| 4 | 8 | 40 | 0.5 | 1.5 | 18.8 | 120 | 1.5 | 60 | 50 |
| 5 | 8 | 40 | 0.5 | 2 | 25 | 120 | 1.5 | 60 | 50 |
| Optimization of Cross-linker | | | | | | | | | |
| 6 | 8 | 40 | 0.5 | 1 | 12.5 | 120 | 1.5 | 60 | 50 |
| 7 | 8 | 80 | 1 | 1 | 12.5 | 120 | 1.5 | 60 | 50 |
| 8 | 8 | 120 | 1.5 | 1 | 12.5 | 120 | 1.5 | 60 | 50 |
| 9 | 8 | 160 | 2 | 1 | 12.5 | 120 | 1.5 | 60 | 50 |
| Optimization of Initiator | | | | | | | | | |
| 10 | 8 | 40 | 0.5 | 1 | 12.5 | 72 | 0.9 | 60 | 50 |
| 11 | 8 | 40 | 0.5 | 1 | 12.5 | 96 | 1.2 | 60 | 50 |
| 12 | 8 | 40 | 0.5 | 1 | 12.5 | 120 | 1.5 | 60 | 50 |
| 13 | 8 | 40 | 0.5 | 1 | 12.5 | 144 | 1.8 | 60 | 50 |
| 14 | 8 | 40 | 0.5 | 1 | 12.5 | 168 | 2.1 | 60 | 50 |
| Optimization of neutralization degree | | | | | | | | | |
| 15 | 8 | 40 | 0.5 | 1 | 12.5 | 120 | 1.5 | 40 | 50 |
| 16 | 8 | 40 | 0.5 | 1 | 12.5 | 120 | 1.5 | 50 | 50 |
| 17 | 8 | 40 | 0.5 | 1 | 12.5 | 120 | 1.5 | 60 | 50 |
| 18 | 8 | 40 | 0.5 | 1 | 12.5 | 120 | 1.5 | 70 | 50 |
| 19 | 8 | 40 | 0.5 | 1 | 12.5 | 120 | 1.5 | 80 | 50 |
| Optimization of water added | | | | | | | | | |
| 20 | 8 | 40 | 0.5 | 1 | 12.5 | 120 | 1.5 | 40 | 20 |
| 21 | 8 | 40 | 0.5 | 1 | 12.5 | 120 | 1.5 | 50 | 30 |
| 22 | 8 | 40 | 0.5 | 1 | 12.5 | 120 | 1.5 | 60 | 40 |
| 23 | 8 | 40 | 0.5 | 1 | 12.5 | 120 | 1.5 | 70 | 50 |
| 24 | 8 | 40 | 0.5 | 1 | 12.5 | 120 | 1.5 | 80 | 60 |

Figure S4. BET N_2_ adsorption-desorption isotherm of (a) FA, and (c) FAWA, and pore size distribution of (b) FA and (d) FAWA

Table S5. BET surface area and average pore diameter of FA and FAWA

| Material | Surface area (m^2^/g) | Average pore diameter (nm) | Pore volume (cc/g) |
| --- | --- | --- | --- |
| FA | 2.07 | 12.5 | 0.0065 |
| FAWA | 0.08 | 273.4 | 0.005 |

Reference

1. Standard, A. S. T. M. C618-19. Standard Specification for Coal Fly Ash and Raw or Calcined Natural Pozzolan for Use in Concrete, ASTM International, West Conshohocken, PA (2011).
